# AI Chatbot Dependence as an Indirect Link Between Social Anxiety and Psychological Resilience: The Moderating Role of Perceived Social Support

**DOI:** 10.3390/bs16091505

**Published:** 2026-08-27

**Authors:** Xiaotong Shen, Changyuan Liu, Jinzhe Yan

**Affiliations:** Department of Business Administration, Gachon University, Seongnam 13120, Republic of Korea; 202355036@gachon.ac.kr

**Keywords:** social anxiety, AI chatbot dependence, psychological resilience, perceived social support, generative artificial intelligence, digital mental health

## Abstract

Generative artificial intelligence has transformed AI chatbots into systems that can provide information, emotional feedback, and social interaction. Although prior research has examined AI adoption, trust, and continuance intention, less is known about sustained reliance on AI chatbots and its relationship with psychological adaptation. Drawing on an integrated compensation–resource framework, this study examined whether social anxiety was indirectly associated with psychological resilience through AI chatbot dependence and whether this association varied across levels of perceived social support. Cross-sectional survey data from 395 adult AI chatbot users recruited through an online survey platform in China were analyzed using regression-based path analysis and PROCESS Model 7. Social anxiety was positively associated with AI chatbot dependence and negatively associated with psychological resilience, while AI chatbot dependence was negatively associated with resilience after accounting for social anxiety. A small but statistically significant negative indirect association was observed through AI chatbot dependence. Higher perceived social support was associated with a weaker social anxiety–dependence association and a smaller negative indirect association. This buffering pattern was statistically significant but modest and weakened rather than eliminated the indirect association. These findings identify a context-specific pattern of statistical associations in human–AI interaction while underscoring the need for longitudinal research to establish temporal and causal direction.

## 1. Introduction

Generative AI is increasingly transforming the ways in which people interact with intelligent systems. AI chatbots such as ChatGPT, Claude, and Gemini are no longer merely information-retrieval tools; they can generate context-sensitive responses, maintain conversational continuity, provide personalized feedback, and simulate social interaction (Dwivedi et al., 2023; Pentina et al., 2023). These characteristics distinguish generative AI from many conventional digital platforms because the same system can simultaneously serve instrumental, informational, and socioemotional functions. Recent organizational research also shows that AI can influence knowledge-management and innovation processes (X. Li et al., 2026), illustrating the breadth of behavioral and organizational consequences now being examined in the expanding AI literature.

Many users now use AI chatbots to express emotions, seek guidance, address everyday problems, and experience a sense of companionship. Prior work indicates that companion chatbots can be experienced as sources of companionship, emotional support, and informational support (Ta et al., 2020), while supportive AI messages can enhance perceived social support and emotional validation (Merrill et al., 2025). More recent evidence suggests that social chatbots may be especially salient for users experiencing social or emotional difficulties: a four-week study found changes in loneliness and social anxiety during repeated social-chatbot use, while qualitative accounts emphasized empathy, accessibility, and nonjudgmental interaction as valued features (Kim et al., 2025). These developments suggest that AI chatbots can become recurring social-interaction partners rather than remaining purely instrumental tools.

Existing research has primarily focused on trust in AI systems (Shin, 2021), users’ adoption and continued use of generative AI technologies (Dwivedi et al., 2023), empirical relationship development with social chatbots (Pentina et al., 2023), and broader conceptual and societal debates concerning human–AI companionship (Andersson, 2025; Malfacini, 2025). Emerging work has also begun to examine problematic AI chatbot use and its psychological correlates. For example, Yao et al. (2025) reported that social anxiety was implicated in problematic AI chatbot use within a broader model of self-esteem and psychological states. Nevertheless, an important conceptual gap remains between problematic use and the broader construct of AI chatbot dependence as sustained reliance on AI for information, emotional support, and everyday decision-making. Understanding this distinction is necessary before stronger claims about maladaptation or harm can be made.

Social anxiety is an important psychological factor shaping technology-mediated interaction. Classic studies linked social anxiety with compensatory Internet use and preferences for lower-risk communication channels (Caplan, 2007; Lee & Stapinski, 2012; Weidman et al., 2012). Recent AI-specific evidence extends this issue by showing that socially anxious users may value the nonjudgmental and controllable features of generative AI and that social anxiety can be associated with problematic AI-chatbot engagement (Kim et al., 2025; Yao et al., 2025). Unlike human-to-human online interaction, AI chatbots are continuously available, responsive on demand, and capable of generating personalized social cues without directly exposing users to interpersonal evaluation. These features make AI chatbots a theoretically distinctive setting in which to examine sustained reliance among socially anxious individuals.

Resilience is treated in this study as a central adaptive resource that helps individuals withstand, recover from, and adjust to stress, setbacks, and adversity (Connor & Davidson, 2003; Southwick et al., 2014). Because AI chatbots can provide both instrumental assistance and socioemotional interaction, greater reliance on them may coexist with either adaptive or maladaptive patterns depending on how such use relates to offline coping and interpersonal resources. Accordingly, the present study does not assume that a high AI chatbot dependence score is inherently problematic. Instead, it examines whether greater reported reliance is statistically associated with psychological resilience and whether perceived support from real interpersonal networks conditions this pattern.

The present study therefore addresses three related questions. First, it examines whether social anxiety is associated with greater AI chatbot dependence in a context where AI provides personalized, low-evaluation, and socially responsive interaction. Second, it tests whether AI chatbot dependence constitutes an indirect statistical link between social anxiety and psychological resilience without treating that cross-sectional link as a temporal or causal mechanism. Third, it examines perceived social support as a potential boundary condition. Using data from 395 adult AI chatbot users, the study positions generative AI not merely as another digital channel but as an interactive artificial social agent whose simultaneous instrumental and socioemotional functions may alter the meaning of technology reliance.

## 2. Literature Review

### 2.1. Theoretical Foundation and Conceptual Boundary

The theoretical framework is organized as a sequential compensation–resource account rather than as four independent theories assigned to separate paths. Social compensation theory and compensatory Internet use theory jointly explain why individuals experiencing difficulty or unmet needs in offline interaction may turn toward lower-risk technological channels. In the present context, these perspectives motivate the association between social anxiety and AI chatbot reliance. Conservation of resources theory then provides a resource-based lens for considering how such reliance may covary with resilience, while the stress-buffering framework specifies perceived social support as a boundary condition that may weaken the initial social anxiety–dependence association. Thus, the four perspectives are complementary in function: compensation theories address technology selection, the resource perspective addresses adaptation-related correlates, and stress buffering addresses conditionality. No single perspective addresses all components of the model: the compensation perspectives explain channel selection, the resource perspective frames adaptation-related covariation, and the stress-buffering perspective explains heterogeneity by interpersonal support.

Social compensation theory suggests that individuals who encounter difficulties in offline social interactions or whose need for belonging is unmet may seek psychological compensation through alternative channels of communication (McKenna & Bargh, 2000). Compensatory Internet use theory develops a closely related logic by emphasizing that technology use can serve coping and need-fulfillment functions when users experience loneliness, anxiety, or social stress (Kardefelt-Winther, 2014). These perspectives are therefore treated here as a common motivational layer rather than as competing explanations. Earlier Internet research linked social anxiety with compensatory digital communication (Caplan, 2007; Lee & Stapinski, 2012; Weidman et al., 2012), and recent AI-specific work similarly suggests that social anxiety is relevant to problematic AI-chatbot engagement (Yao et al., 2025).

Generative AI changes this compensatory setting in several ways. Unlike many conventional platforms, a chatbot can respond immediately, adapt its language to the user, preserve conversational context, generate interpersonal cues, and provide informational and emotional assistance within the same interaction. Social chatbots can therefore be experienced as artificial social agents as well as tools (Kim et al., 2025; Pentina et al., 2023). This combination may be particularly attractive when a user wishes to obtain support or interaction while minimizing exposure to negative evaluation. The theoretical contribution is not that compensation is unique to AI, but that generative AI concentrates instrumental utility and quasi-social responsiveness in a single, continuously available interaction partner. Psychologically, this may allow instrumental help, socioemotional reassurance, and avoidance of interpersonal evaluation to occur within the same interaction, thereby changing the object and form of reliance.

A further conceptual distinction is necessary. In this study, AI chatbot dependence refers to the degree of sustained reliance captured by the AICDS, including reliance for information acquisition, emotional support, and everyday decision-making (Zhang et al., 2025). Dependence in this operational sense is not synonymous with frequent use, habitual use, productive reliance, addiction, or problematic use. A person may use an AI chatbot intensively for legitimate instrumental reasons without experiencing maladaptation, whereas problematic use generally implies impaired control, functional interference, or adverse consequences (Yao et al., 2025). Accordingly, higher AICDS scores are interpreted as greater reliance rather than as evidence that every high-scoring user has a harmful or pathological relationship with AI.

Conservation of resources theory provides a complementary lens for examining how differences in reliance may be associated with psychological adaptation. People seek to obtain and preserve resources that support coping and adjustment (Hobfoll, 1989), and resilience reflects the capacity to mobilize psychological, interpersonal, and social resources under adversity (Connor & Davidson, 2003; Southwick et al., 2014). AI-based assistance may itself function as a resource, but if greater reliance coincides with fewer opportunities or lower motivation to draw on autonomous coping or interpersonal resources, it may be associated with lower resilience. The stress-buffering framework adds a boundary condition: stronger perceived support from close others may reduce the need to use AI as a compensatory social channel (Cohen & Wills, 1985). These propositions concern statistical covariation in the present cross-sectional study and do not establish a developmental sequence.

### 2.2. Social Anxiety and AI Chatbot Dependence

Social anxiety refers to persistent tension, avoidance, and discomfort in social situations arising from fear of negative evaluation (Clark & Wells, 1995). Socially anxious individuals often perceive face-to-face interaction as more threatening and may prefer communication channels that reduce evaluative pressure and increase controllability (Alden & Taylor, 2004). Although foundational evidence comes from pre-generative-AI contexts, recent work confirms the continuing relevance of social anxiety in AI-mediated settings. Kim et al. (2025) examined social anxiety during repeated social-chatbot interaction, while Yao et al. (2025) identified social anxiety as a psychological factor associated with problematic AI chatbot use.

Within the compensation perspective, the relevant issue is not simply screen time but the affordances of the interaction channel. Generative AI chatbots are available on demand, do not directly judge the user, allow the user to control conversational pacing, and can produce socially responsive and personalized replies. These affordances may make AI interaction particularly appealing to users who experience greater discomfort or uncertainty in interpersonal settings. Thus, the extension from earlier digital-dependence research lies in examining reliance on a responsive artificial social agent rather than only on a communication platform connecting human users.

At the same time, greater use frequency should not be equated with greater AI chatbot dependence as operationalized here. Daily usage duration is therefore treated as a distinct behavioral characteristic and reported descriptively rather than as a proxy for dependence. The focal construct instead concerns the extent to which users report relying on chatbots for information, emotional support, and decisions. On this basis, socially anxious individuals are expected to report greater AI chatbot dependence, without implying that such dependence is necessarily problematic. Therefore, this study proposes:

**H1.**  *Social anxiety is positively associated with AI chatbot dependence.*

### 2.3. Social Anxiety, AI Chatbot Dependence, and Resilience

Psychological resilience refers to the capacity to maintain or recover adaptive functioning when facing stress, adversity, and challenges (Connor & Davidson, 2003). Resilience depends on personal and contextual resources, including coping capacities, interpersonal ties, and social support (Luthar et al., 2000; Southwick et al., 2014). Social anxiety is consistently associated with difficulties in social interaction and with reduced access to some interpersonal resources. Recent evidence among college students also reports an inverse association between trait resilience and social anxiety (Y. Li & Zheng, 2025). Accordingly, the present study expects higher social anxiety to be associated with lower psychological resilience, while recognizing that the cross-sectional design cannot determine whether one precedes the other. Therefore, this study proposes:

**H2.**  *Social anxiety is negatively associated with psychological resilience.*

AI chatbots can provide informational assistance, emotional feedback, and perceived social support, and recent research suggests that social-chatbot use may sometimes accompany reductions in loneliness or social anxiety (Kim et al., 2025), while experimentally manipulated supportive chatbot messages can enhance perceived emotional validation and social support (Merrill et al., 2025). These findings caution against treating AI reliance as uniformly harmful. At the same time, dependence as measured here reflects the extent to which a user reports relying on AI across multiple everyday functions. Such reliance may be negatively associated with resilience when it co-occurs with lower autonomous coping or reduced engagement with interpersonal resources, but the present data cannot identify those processes directly.

Research on problematic Internet, social-media, and smartphone use has often reported associations with poorer mental-health outcomes (Elhai et al., 2016; Marino et al., 2018), while emerging AI-specific studies distinguish beneficial support from problematic or excessive use (Kim et al., 2025; Yao et al., 2025). This distinction is important for interpreting the AICDS: the current study tests whether greater reliance is associated with resilience, not whether ordinary or productive AI use is intrinsically detrimental. Accordingly, the expected negative association should be understood as a population-level statistical relationship rather than evidence that reliance on AI chatbots causes a reduction in resilience. Therefore, this study proposes:

**H3.**  *AI chatbot dependence is negatively associated with psychological resilience.*

Taken together, the compensation and resource perspectives motivate an indirect statistical pattern. Social anxiety may be associated with greater reliance on a low-evaluation and controllable artificial interaction partner, while greater AI chatbot dependence may in turn be statistically associated with lower resilience. Because all variables are measured concurrently, this proposed ordering is theory-guided rather than temporally demonstrated. AI chatbot dependence is therefore treated as an indirect statistical link between social anxiety and psychological resilience, not as evidence of a causal mediating mechanism. Therefore, this study proposes:

**H4.**  *Social anxiety is indirectly and negatively associated with psychological resilience through AI chatbot dependence.*

### 2.4. The Moderating Role of Perceived Social Support

In the present study, perceived social support denotes individuals’ belief that supportive resources can be obtained through close social ties, including relatives, peers, and important relationship partners (Zimet et al., 1988). The stress-buffering framework proposes that supportive interpersonal resources can reduce the strain associated with stress-related vulnerability and facilitate coping (Cohen & Wills, 1985). In the integrated framework, this perspective is used specifically to explain why the association between social anxiety and compensatory AI reliance may differ across levels of offline interpersonal support.

Among individuals with social anxiety, stronger interpersonal support may satisfy some needs for belonging, emotional reassurance, and practical assistance outside the chatbot context (Taylor, 2011). Prior studies have associated social support with resilience and adaptive coping (Ozbay et al., 2007), while research on online and offline support suggests that technologically mediated support does not necessarily substitute for support received from close interpersonal relationships (Longest & Kang, 2022). This logic implies that greater perceived support may reduce, rather than eliminate, the statistical association between social anxiety and AI chatbot dependence.

This proposition does not assume that AI support and interpersonal support are functionally equivalent or that one necessarily displaces the other. Rather, perceived social support is treated as a contextual resource that may change the extent to which socially anxious users report relying on AI for compensatory functions. Because any moderation observed in cross-sectional data is associational, both statistical significance and substantive magnitude must be evaluated.

Accordingly, the study proposes the following conditional indirect hypothesis:

**H5.**  *The indirect association between social anxiety and psychological resilience through AI chatbot dependence is conditional on perceived social support, such that the negative indirect association is weaker at higher levels of perceived social support.*

**Figure 1:**
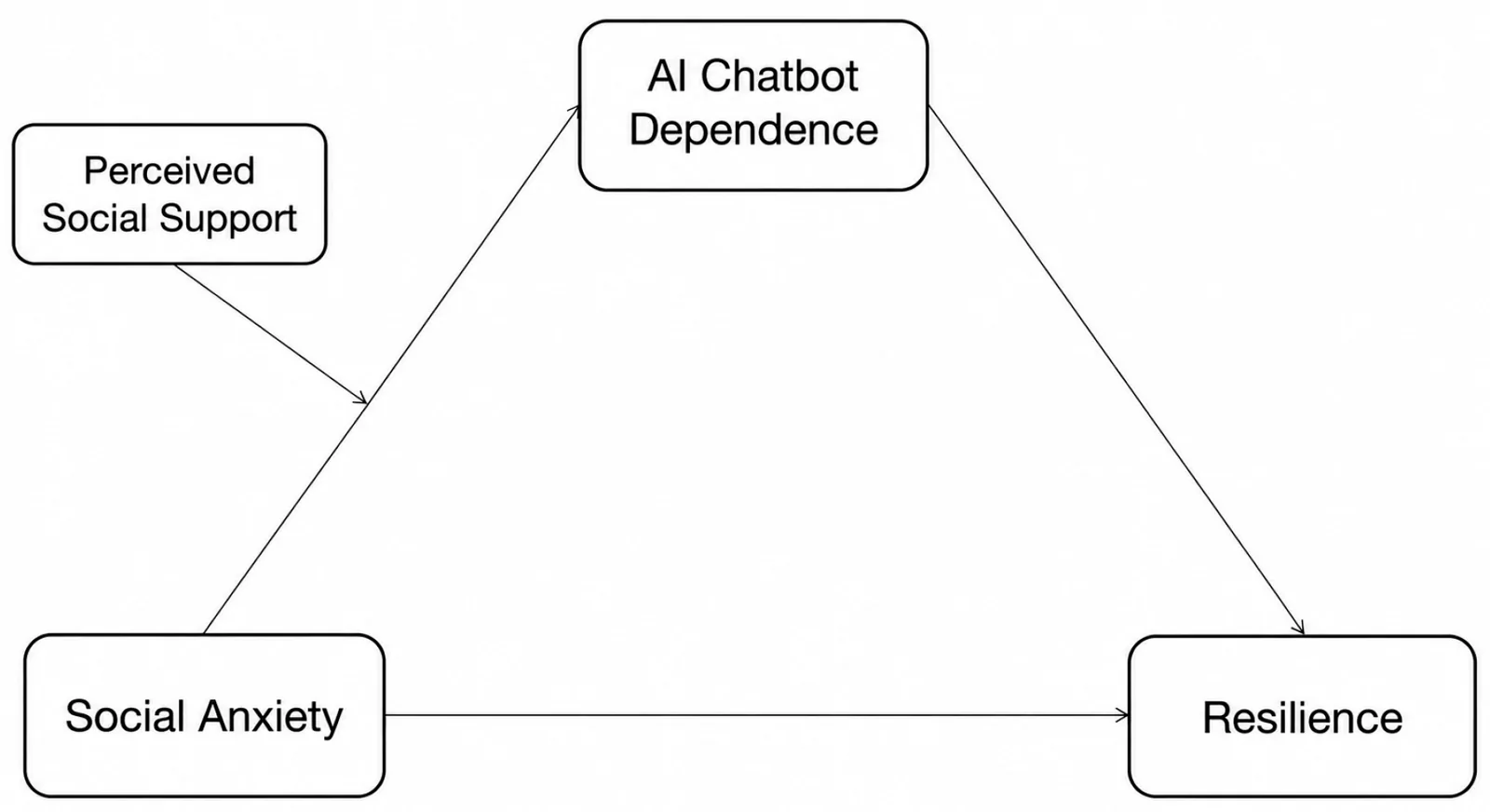
Research Model.

Taken together, H1–H3 specify the three focal pairwise associations, H4 specifies the corresponding indirect statistical association, and H5 specifies whether that indirect association varies by perceived social support. Figure 1 summarizes these theory-guided associations; none of the hypotheses is intended to establish temporal or causal ordering from the cross-sectional design.

The proposed research model is shown in Figure 1.

## 3. Materials and Methods

### 3.1. Sample and Data Collection

The survey was conducted on the Wenjuanxing platform using its sample-recruitment service in China. Participants received RMB 7 after completing the questionnaire. Prior to participation, respondents read an information notice describing the study aims, academic use of the data, anonymous data handling, privacy-protection procedures, voluntary participation, and the right to stop before submission without penalty. They then provided electronic informed consent and answered a screening question asking whether they had used an AI chatbot such as ChatGPT, Claude, Gemini, or Character.AI during the previous six months. Those selecting “No” were directed to the end of the survey. A total of 405 response records were initially obtained. Following data screening, 10 responses were excluded: two because the respondents did not meet the AI chatbot usage criterion, six because the questionnaires were completed in less than 1 min, one because of straight-line responding across all scale items, and one because the respondent was younger than 18 years and the study’s consent procedure did not include parental or guardian consent. The final analytic sample consisted of 395 adult participants (97.53% of the initially obtained records). Wenjuanxing also retained IP-based geolocation at the time of questionnaire submission. Of the final records, 393 were geolocated to 27 provincial-level regions in mainland China and two were associated with overseas IP locations. Because IP geolocation does not necessarily represent a respondent’s permanent or usual residence, these data were used only to describe sample distribution and were not treated as self-reported residence.

### 3.2. Measures

The questionnaire consisted of three sections: informed consent and eligibility screening, demographic and AI chatbot usage information, and the measurement scales. Demographic and background information included gender, age, educational level, and average daily AI chatbot usage. Gender was categorized as male or female. Age was recorded as under 18, 18–25, 26–35, 36–45, or 46 years or above; the single respondent younger than 18 was excluded as described in Section 3.1. Educational attainment was recorded as high school level or lower, junior college, bachelor’s degree, master’s degree, or doctoral level or higher. Average daily AI chatbot use was grouped into five time bands: under 30 min, 30 min–1 h, 1–2 h, 2–4 h, and over 4 h. The main constructs were operationalized with previously validated instruments. Respondents answered scale items on a seven-category agreement format from 1 (strongly disagree) to 7 (strongly agree). Social anxiety was captured with the SIAS-6 (Peters et al., 2012). AI chatbot dependence was measured using the eight-item AICDS developed by Zhang et al. (2025), which assesses reliance on AI chatbots for information acquisition, emotional support, and everyday decision-making. Consistent with the conceptual boundary described in Section 2.1, AICDS scores were interpreted as degree of reliance rather than as evidence of addiction or problematic use. Perceived social support was assessed with the 12-item MSPSS (Zimet et al., 1988), and psychological resilience was operationalized with the CD-RISC-10 (Campbell-Sills & Stein, 2007). All measurement items are appended in the Supplementary Material.

### 3.3. Data Analysis Procedure

The empirical analysis was conducted with SPSS 29.0, AMOS 26.0, and PROCESS Macro 4.2. Consistent with Anderson and Gerbing’s (1988) two-stage logic, construct measurement was examined first, followed by tests of the proposed associations. The analysis began with frequency distributions, descriptive statistics, and Pearson correlations. Potential common method bias was assessed with Harman’s single-factor diagnostic and full-collinearity VIFs; these procedures were treated as diagnostic checks rather than tests capable of demonstrating the absence of common method bias. AMOS 26.0 was then used to estimate the confirmatory factor model. Reliability and convergent-validity evidence were examined through Cronbach’s α, CR, AVE, item loadings, corrected item–total correlations, and α if item deleted, whereas discriminant validity was evaluated with the Fornell–Larcker criterion and HTMT ratios.

Regression-based path analysis using observed composite scores was then used to estimate the focal associations. AI chatbot dependence was regressed on social anxiety, and psychological resilience was regressed on social anxiety and AI chatbot dependence. The indirect association through AI chatbot dependence was evaluated using 5000 bootstrap resamples (Preacher & Hayes, 2008). Hayes’s PROCESS Model 7 was used for the conditional indirect analysis, with perceived social support moderating the social anxiety–AI chatbot dependence association. Incremental explanatory power was also evaluated hierarchically by entering social anxiety, perceived social support, and their mean-centered interaction in successive steps. Thus, H1–H3 were evaluated with the regression paths, H4 with the bootstrap indirect association, and H5 with PROCESS Model 7. An indirect or conditional indirect association was considered statistically supported when its 95% bootstrap confidence interval did not include zero. Because all focal variables were measured at a single time point, these estimates represent statistical associations and do not establish temporal ordering or causality.

Average daily AI chatbot usage was reported as a descriptive behavioral characteristic but was not entered as a routine covariate in the hypothesis-testing models. Usage duration and AICDS scores were measured concurrently and capture related but non-equivalent aspects of AI engagement: behavioral exposure versus broader functional and self-reported reliance. In the present cross-sectional design, daily usage cannot be established as a temporally prior confounder; routine adjustment for a conceptually proximal concurrent variable could remove theoretically meaningful variance without resolving temporal ambiguity. Consistent with recommendations that statistical controls should be theoretically justified rather than entered automatically (Bernerth & Aguinis, 2016), gender, age, and education were likewise reported descriptively because the theoretical model did not specify them a priori as common causes of the focal associations. Accordingly, the analyses were kept theory-driven and parsimonious. Future longitudinal research should separate usage intensity and dependence temporally and pre-specify demographic or behavioral covariates when their confounding role is theoretically justified.

## 4. Results

### 4.1. Respondent Profile

Of the 405 initially obtained response records, 10 were excluded following the screening procedures described in Section 3.1. The final analytic sample consisted of 395 adult participants (97.53%). Table 1 reports gender, age, educational background, daily AI chatbot usage, and the descriptive distribution of IP-based geographic locations. The sample was concentrated in Eastern China but included submissions from all four broad mainland regions; two records were associated with overseas IP locations. These geographic data describe where the questionnaire connection was geolocated and should not be interpreted as verified permanent residence.

The respondent profile therefore showed variation in gender, age, daily AI use, and geographic connection location, although the sample was heavily concentrated among respondents with bachelor’s degrees. These distributions are reported descriptively and do not imply population representativeness.

### 4.2. Common Method Bias

Given the use of cross-sectional self-report data, potential common method bias was assessed before hypothesis testing. In the Harman single-factor diagnostic, the largest unrotated factor accounted for 46.183% of the total variance; thus, no single factor accounted for a majority of the variance. Consistent with the caution emphasized by Podsakoff et al. (2003), we treated this result only as a diagnostic indication, not as evidence that common method bias was absent. Full-collinearity VIFs ranged from 1.698 to 2.650, below the 3.3 criterion proposed by Kock (2015). These diagnostics did not indicate severe common method problems. However, neither Harman’s test nor full-collinearity VIFs can establish that common method bias is absent. Because all focal constructs were self-reported by the same respondents at one time point, residual common method bias remains possible and is treated as a study limitation (Table 2).

### 4.3. Measurement Model Assessment

#### 4.3.1. Reliability and Convergent Validity

For the four constructs, reliability and convergence evidence were evaluated through Cronbach’s α, composite reliability (CR), average variance extracted (AVE), and item loadings. Nunnally and Bernstein’s (1994) reliability guideline set 0.700 as the minimum acceptable Cronbach’s α value. CR values above 0.700 and AVE values above 0.500 were used as the criteria for establishing composite reliability and convergent validity, respectively (Fornell & Larcker, 1981).

Table 3 shows strong scale-level reliability: Cronbach’s α ranged from 0.905 to 0.952, and CR ranged from 0.905 to 0.952, with all values above 0.700. Because Cronbach’s α, CR, and AVE are construct-level rather than item-level indices, they are reported once for each construct. To provide item-level reliability information, corrected item–total correlations and Cronbach’s α if items are deleted are reported separately in Table 4.

The AVE values ranged from 0.545 to 0.664. Specifically, the AVE values were 0.657 for social anxiety, 0.545 for AI chatbot dependence, 0.567 for perceived social support, and 0.664 for psychological resilience. All values exceeded the recommended threshold of 0.500, providing evidence of satisfactory convergent validity.

Standardized item loadings ranged from 0.643 to 0.849 and were statistically significant at *p* < 0.001. Corrected item–total correlations ranged from 0.629 to 0.822, and α-if-item-deleted values remained high across all items, indicating that no single item disproportionately determined scale reliability. Together with construct-level CR and AVE, these results support acceptable internal consistency and convergence without treating CR or AVE as item-level statistics.

#### 4.3.2. Confirmatory Factor Analysis

To further assess construct validity, a confirmatory factor model was estimated in AMOS 26.0 (Byrne, 2016). Model fit was evaluated using χ^2^/*df*, CFI, TLI, RMSEA, and SRMR. Fit was interpreted from the overall pattern of indices and commonly used guidelines rather than as a set of rigid universal cutoffs (Hu & Bentler, 1999; Kline, 2023). As shown in Table 5, the overall pattern of fit indices indicated an acceptable fit of the measurement model. The CFA results therefore support the construct validity of the measurement model.

#### 4.3.3. Discriminant Validity

Discriminant validity was further examined to ensure that the latent variables represented theoretically distinct constructs. As the first check, discriminant validity was evaluated using the AVE square-root comparison proposed by Fornell and Larcker (1981). Under this rule, each latent variable’s AVE square root should exceed its associations with the remaining latent variables. As shown in Table 6, the square roots of the AVE for social anxiety, AI chatbot dependence, perceived social support, and psychological resilience were all greater than their inter-construct correlations, supporting discriminant validity.

For an additional check, HTMT ratios were inspected because Henseler et al. (2015) recommend them for SEM-based discriminant validity assessment. Ratios that do not reach 0.850 indicate sufficient separation among latent variables. Table 7 shows that the ratios ranged from 0.521 for AI chatbot dependence and perceived social support to 0.767 for social anxiety and resilience, all within the criterion. The constructs were therefore empirically distinct.

The results of Cronbach’s α, CR, AVE, CFA model fit, the Fornell–Larcker criterion, and HTMT indicate that the measurement model met the recommended standards for reliability, convergent validity, construct validity, and discriminant validity. Therefore, the model was suitable for subsequent regression-based hypothesis testing.

### 4.4. Descriptive Statistics and Correlations

Table 8 reports descriptive statistics and Pearson correlations for the main variables. Social anxiety was positively correlated with AI chatbot dependence (*r* = 0.623, *p* < 0.001), indicating that respondents with higher levels of social anxiety tended to report greater dependence on AI chatbots. This result is consistent with social compensation theory and compensatory internet use theory and provides preliminary support for H1.

Social anxiety was negatively correlated with psychological resilience (*r* = −0.718, *p* < 0.001), consistent with H2. AI chatbot dependence was also negatively correlated with psychological resilience (*r* = −0.525, *p* < 0.001), although this association was weaker than the correlation between social anxiety and resilience. Perceived social support showed a positive correlation with resilience (*r* = 0.680, *p* < 0.001). These bivariate associations were examined further in the regression analyses.

Overall, the correlation results were consistent with theoretical expectations. No correlation coefficient exceeded 0.800, and the regression diagnostics reported above did not indicate severe multicollinearity.

### 4.5. Regression Results

Regression-based path analysis using observed composite scores was used to test H1–H3. As shown in Table 9, social anxiety was positively associated with AI chatbot dependence (*β* = 0.623, *b* = 0.601, *t* = 15.770, *p* < 0.001). This finding indicates that individuals with higher levels of social anxiety tended to report greater reliance on AI chatbots for information retrieval, emotional regulation, and problem solving in daily life. Thus, H1 was supported.

Social anxiety was negatively associated with psychological resilience (*β* = −0.640, *b* = −0.573, *t* = −14.396, *p* < 0.001). The negative coefficient indicates that respondents with higher levels of social anxiety tended to report lower psychological resilience. Thus, H2 was supported.

After accounting for social anxiety, AI chatbot dependence remained negatively associated with psychological resilience (*β* = −0.126, *b* = −0.117, *t* = −2.843, *p* = 0.005). Thus, H3 was supported.

Regarding explanatory power, the model predicting AI chatbot dependence accounted for 38.8% of the variance (*R*^2^ = 0.388), and the model predicting psychological resilience accounted for 52.6% (*R*^2^ = 0.526). Effect-size estimates were large for the social-anxiety associations with AI chatbot dependence (*f*^2^ = 0.633) and resilience (*f*^2^ = 0.529), whereas the unique association between AI chatbot dependence and resilience was small (*f*^2^ = 0.021). Table 8 reports *b*, *β*, *t*, *p*, confidence intervals, *R*^2^, and *f*^2^ to make the hypothesis tests and explanatory power more explicit.

### 4.6. Indirect Association Analysis

To examine the indirect association between social anxiety and psychological resilience through AI chatbot dependence, the bootstrap method with 5000 resamples was used. As shown in Table 10, social anxiety was positively associated with AI chatbot dependence (a = 0.601, *p* < 0.001), whereas AI chatbot dependence was negatively associated with psychological resilience after accounting for social anxiety (*b* = −0.117, *p* = 0.005). Thus, both component associations of the estimated indirect association were statistically significant.

The estimated indirect association was −0.070, and its 95% bootstrap confidence interval ranged from −0.120 to −0.022. Because the interval did not include zero, the indirect association was statistically significant, providing support for H4. The direct association between social anxiety and psychological resilience also remained statistically significant after accounting for AI chatbot dependence (*b* = −0.573, 95% CI [−0.655, −0.489]). Thus, the results indicate a statistically significant indirect association alongside a remaining direct association.

Together, these estimates are consistent with the hypothesized indirect pattern linking social anxiety, AI chatbot dependence, and psychological resilience. Given the cross-sectional design, however, the results do not establish temporal ordering or a causal mechanism.

### 4.7. Conditional Indirect Association Analysis

Finally, the conditional indirect hypothesis was examined with Hayes’s (2022) PROCESS Model 7. Model 7 is appropriate when the moderator operates on the first-stage path of the mediation model. In this study, perceived social support was specified as the moderator of the path from social anxiety to AI chatbot dependence, thereby shaping the indirect association between social anxiety and psychological resilience through AI chatbot dependence.

As shown in Table 11, social anxiety was positively associated with AI chatbot dependence (*b* = 0.489, *t* = 10.598, *p* < 0.001), whereas perceived social support was negatively associated with AI chatbot dependence (*b* = −0.171, *t* = −2.759, *p* = 0.006). More importantly, the Social Anxiety × Perceived Social Support interaction was statistically significant (*b* = −0.066, *t* = −2.134, *p* = 0.033). This pattern indicates that the positive association between social anxiety and AI chatbot dependence was weaker at higher levels of perceived social support.

To evaluate incremental explanatory power within the same observed-score regression framework, the predictors were entered hierarchically. Social anxiety alone accounted for 38.8% of the variance in AI chatbot dependence (*R*^2^ = 0.388). Adding perceived social support increased *R*^2^ to 0.409 (Δ*R*^2^ = 0.021, Δ*F*(1, 392) = 14.09, *p* < 0.001). Adding the Social Anxiety × Perceived Social Support interaction further increased *R*^2^ to 0.416 (Δ*R*^2^ = 0.007, Δ*F*(1, 391) = 4.56, *p* = 0.033). Thus, the interaction term accounted for a small but statistically significant increment in explained variance.

**Figure 2:**
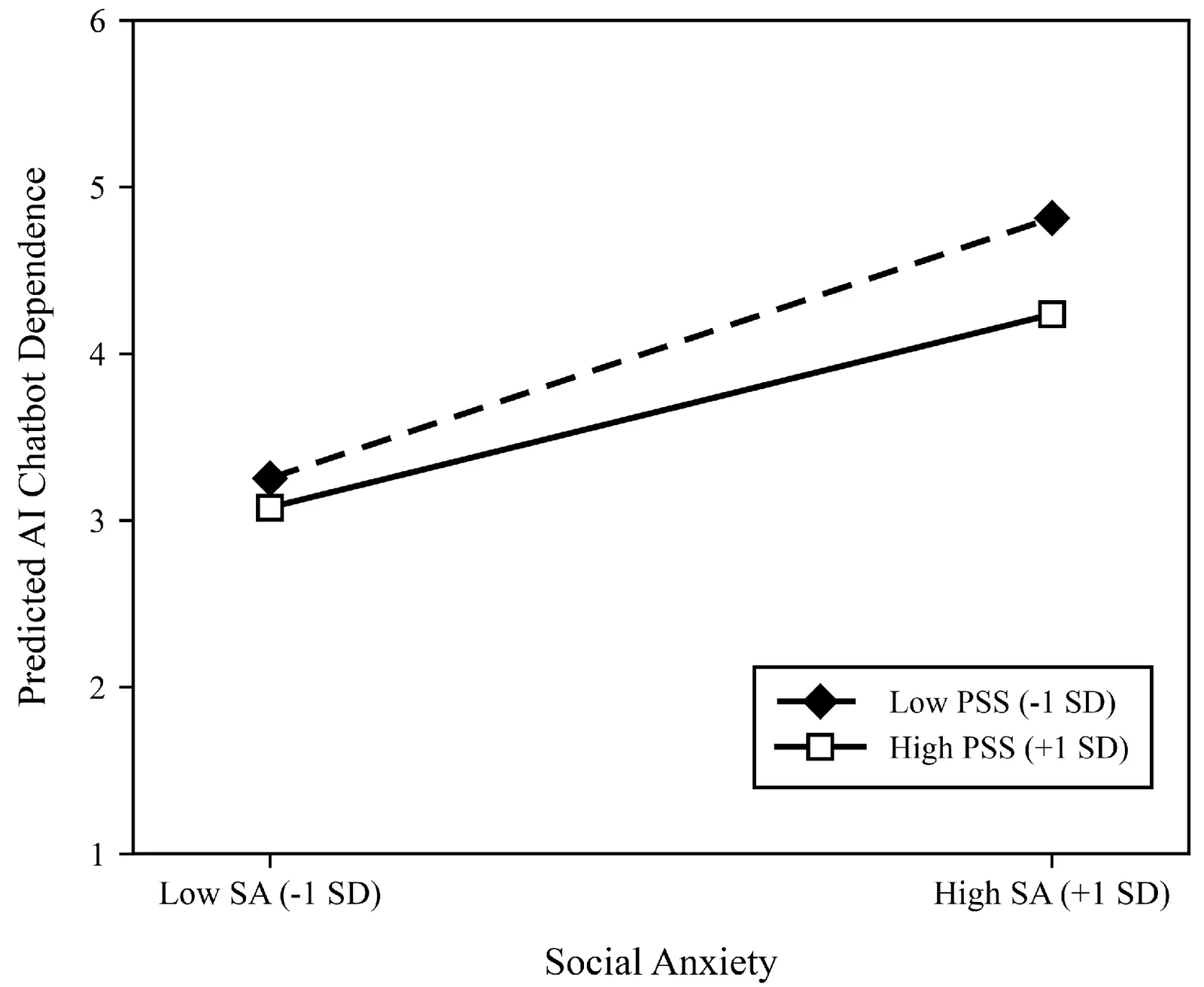
Interaction Effect.

Conditional indirect associations were estimated at low, average, and high levels of perceived social support (PSS). At −1 *SD* of PSS, the estimated conditional indirect association was −0.066, 95% CI [−0.107, −0.022]. At the mean level of PSS, the estimate was −0.057, 95% CI [−0.094, −0.019]. At +1 *SD* of PSS, the estimate was −0.049, 95% CI [−0.083, −0.015]. Figure 2 illustrates the interaction effect. Because none of the 95% bootstrap confidence intervals included zero, the conditional indirect association was statistically different from zero at all three levels of perceived social support.

The index of moderated mediation was 0.008, 95% CI [0.001, 0.016]. Because the confidence interval did not include zero, the magnitude of the indirect association varied significantly across levels of perceived social support, supporting H5. Table 12 reports the corresponding conditional indirect associations and index of moderated mediation.

## 5. Discussion

### 5.1. Interpretation of Findings

The findings identify a conditional pattern of associations among social anxiety, AI chatbot dependence, psychological resilience, and perceived social support. The results should be interpreted as cross-sectional statistical relationships rather than as a developmental process. Three aspects are especially important for interpretation: the generative-AI context changes the object of reliance from a general digital medium to a responsive artificial social agent; AI chatbot dependence in the AICDS sense denotes sustained reliance rather than necessarily problematic use; and the perceived-social-support moderation was statistically significant but modest in substantive magnitude.

First, higher social anxiety was associated with greater AI chatbot dependence. This result is consistent with social compensation and compensatory Internet use perspectives, but it also aligns with more recent AI-specific evidence. Yao et al. (2025) found that social anxiety was implicated in problematic AI chatbot use, while Kim et al. (2025) reported reductions in social anxiety over four weeks of social-chatbot use, and qualitative accounts emphasized empathy, accessibility, and nonjudgmental interaction. The present result differs from classic Internet findings in the object of reliance: generative chatbots can combine instrumental assistance with personalized quasi-social interaction, allowing socially anxious users to seek information, guidance, and socioemotional interaction from the same artificial agent.

Second, social anxiety was negatively associated with psychological resilience. This finding is consistent with recent evidence of an inverse association between trait resilience and social anxiety among college students (Y. Li & Zheng, 2025) and with broader resource-based accounts of adaptation. Nevertheless, the present cross-sectional data do not show that social anxiety reduces resilience. Lower resilience could also precede greater social anxiety, and both may reflect shared psychological or contextual influences. The result is therefore best understood as evidence that social anxiety and resilience are strongly inversely related in this sample.

Third, AI chatbot dependence showed a small negative association with psychological resilience after accounting for social anxiety. This result should not be generalized to all intensive or productive AI use. Recent work illustrates both potentially supportive and problematic forms of chatbot engagement: social chatbots may provide perceived support and accompany reductions in loneliness or social anxiety (Kim et al., 2025), whereas problematic AI chatbot use has been associated with adverse psychological states (Yao et al., 2025). The present AICDS measure lies conceptually between simple usage frequency and explicitly problematic use. Accordingly, the observed negative association indicates that greater reliance covaried with lower resilience in this sample; it does not demonstrate that AI reliance itself undermines coping resources.

Fourth, the hypothesized negative indirect association through AI chatbot dependence was statistically significant. This finding indicates that the covariance between social anxiety and resilience can be statistically decomposed into a component involving AI chatbot dependence. However, the cross-sectional design remains compatible with plausible reverse or reciprocal relationships. For example, lower resilience may be associated with greater AI reliance, and AI chatbot dependence and social anxiety may reinforce one another. The indirect estimate should therefore be interpreted as a theory-guided statistical association rather than evidence of temporal mediation. Longitudinal or experimental data are required to distinguish competing directional explanations.

Finally, perceived social support weakened the social anxiety–dependence association, and the corresponding indirect association became smaller in absolute magnitude at higher levels of support. However, the substantive magnitude of this moderation was modest. The interaction accounted for only a small increment in explained variance (Δ*R*^2^ = 0.007), and the conditional indirect association remained statistically different from zero at low, mean, and high levels of perceived social support. Accordingly, perceived social support should be interpreted as weakening, rather than eliminating, the indirect association. This pattern is consistent with the stress-buffering framework, but its practical importance should not be overstated.

### 5.2. Theoretical Implications

First, the study specifies what is theoretically distinctive about AI chatbot dependence rather than treating generative AI as a simple relabeling of earlier Internet or smartphone dependence. The underlying compensatory motive is not new; what changes is the technological object. Generative AI combines continuous availability, context-sensitive dialogue, personalized responses, instrumental assistance, and quasi-social interaction in a single artificial agent. This combination can allow the same system to function simultaneously as tool, information source, decision aid, and social-interaction partner. The contribution is therefore to extend compensation-based reasoning to a form of reliance directed toward an artificial social agent with both utilitarian and socioemotional affordances.

Second, the study sharpens the conceptual boundary of AI chatbot dependence. Emerging research increasingly distinguishes problematic AI chatbot use from broader reliance on AI systems (Yao et al., 2025; Zhang et al., 2025). The present findings show that an AICDS score should not automatically be interpreted as addiction, maladaptation, or harm. By separating dependence from daily usage intensity and reporting the latter as a distinct descriptive behavioral characteristic, the study keeps frequency and reliance conceptually and analytically distinct. This distinction is important for future AI-behavior research because intensive use may be productive, habitual, compensatory, or problematic depending on control, function, and consequences.

Third, the integrated theoretical framework is more parsimonious than treating four theories as independent explanations. Social compensation theory and compensatory Internet use theory jointly specify a motivational layer linking social difficulty with lower-risk technology use; the conservation-of-resources perspective provides a lens for interpreting associations with resilience; and stress-buffering theory specifies a potential interpersonal boundary condition. This sequence clarifies why the theories are complementary and also identifies the relative strength of the evidence: the focal associations were statistically supported, whereas the buffering effect was significant but small in incremental explanatory power.

Finally, the results contribute to the growing literature on the behavioral consequences of AI use by showing the importance of analyzing not only adoption and performance outcomes but also the form and function of reliance. Broader AI research has demonstrated that AI can influence knowledge and organizational processes (X. Li et al., 2026), while human–AI research increasingly examines companionship, support, and problematic engagement at the individual level (Kim et al., 2025; Yao et al., 2025). The present study connects these developments by emphasizing that the consequences associated with AI use may depend on how users incorporate AI into their psychological and social resource environments.

These implications remain associational. The data do not establish that AI support displaces interpersonal support, that AI reliance depletes resources, or that offline support dynamically complements AI support over time. Those mechanisms remain theoretically plausible questions for longitudinal and experimental research rather than conclusions of the present study.

### 5.3. Practical Implications

The practical implications should be interpreted as cautious directions for attention rather than as interventions validated by this study. For universities, organizations, and mental-health services, the results indicate that social anxiety, AI chatbot reliance, perceived social support, and resilience can be meaningfully related in AI-using populations. Screening or educational initiatives may therefore benefit from asking not only how often people use AI chatbots but also what functions the systems serve in their daily lives. The present study did not test psychotherapy, counseling, or behavioral interventions, so no specific clinical treatment can be recommended on the basis of these data. This cautious interpretation is consistent with recent evidence that social chatbots can provide supportive experiences in some contexts without implying uniformly beneficial or harmful outcomes (Kim et al., 2025; Merrill et al., 2025).

For AI developers, the findings provide a rationale for considering digital well-being in product design, particularly for features that encourage prolonged or highly dependent use. However, the data do not establish that usage reminders, dependence-risk monitoring, or offline-interaction prompts improve psychological outcomes. Such features should therefore be treated as design hypotheses that require prospective evaluation rather than as evidence-based prescriptions derived from the current study.

For educational institutions and organizations, the statistically significant but modest buffering pattern suggests that interpersonal support may be relevant when considering AI reliance; however, the small incremental explanatory contribution of the interaction warrants caution. Peer support, mentoring, and employee-assistance programs have independent rationales in the broader literature, yet this study does not demonstrate that implementing these programs will reduce AI chatbot dependence or increase resilience. Future intervention studies could test whether strengthening interpersonal support changes patterns of AI reliance over time. This emphasis is also consistent with broader evidence linking perceived social support with resilience and adaptive coping (Ozbay et al., 2007), while the intervention question remains to be tested directly.

For policymakers and AI-governance bodies, the results support continued attention to the psychological and social dimensions of human–AI interaction alongside technical safety and privacy. They do not, however, justify a specific regulatory measure. More direct evidence from longitudinal, experimental, and population-representative studies is needed before translating associations between AI reliance and psychological outcomes into clinical or regulatory requirements.

### 5.4. Limitations and Future Research

First, all focal variables were measured at one time point. Temporal ordering therefore cannot be established, and the significant indirect association should not be interpreted as causal mediation. Plausible reverse or reciprocal relationships remain possible; for example, lower resilience may be associated with greater AI reliance, and AI reliance and social anxiety may reinforce one another. Future studies should use longitudinal cross-lagged designs, intensive repeated measurement, or experiments to compare these competing directional accounts.

Second, participants were recruited through Wenjuanxing’s sample service in China. IP-based submission locations covered 27 mainland provincial-level regions, but the distribution was uneven and two records were associated with overseas IP locations. Because IP geolocation is not equivalent to self-reported residence and may be affected by network routing or travel, the study cannot make strong claims about regional differences. Future research should collect respondents’ usual province or region directly and use stratified or population-based sampling to test geographic heterogeneity. Cross-cultural evidence is also needed to assess generalizability beyond this sample.

Third, AI chatbot dependence was measured with a unidimensional scale emphasizing reliance for information, emotional support, and decision-making. Although item-level and construct-level reliability were satisfactory, the construct does not by itself distinguish productive reliance, habitual use, compulsive use, and problematic engagement. Future work should compare multidimensional measures that separate functional reliance from impaired-control or harm-related dimensions and should examine how those dimensions relate differently to resilience and well-being.

Fourth, the study relied on self-reported measures from the same respondents. Harman’s single-factor test and full-collinearity VIFs did not indicate severe common method problems, but these diagnostics cannot rule out common method bias. Future studies should combine self-report data with behavioral usage logs, informant reports, or temporally separated measurements. In addition, daily AI chatbot usage and demographic characteristics were reported descriptively rather than entered as routine covariates because their role as confounders was not established a priori within the present cross-sectional model; future longitudinal studies should pre-specify and test such covariates under clearer temporal assumptions. Finally, the moderation involving perceived social support was statistically significant but modest in magnitude; replication in independent samples is needed before strong conclusions are drawn about its buffering role.

## 6. Conclusions

In this sample, social anxiety was positively associated with AI chatbot dependence and negatively associated with psychological resilience, while greater AI chatbot dependence was associated with lower resilience after accounting for social anxiety. A statistically significant negative indirect association through AI chatbot dependence was observed. H5 was also supported: higher perceived social support was associated with a weaker social anxiety–dependence association and a smaller negative indirect association. The buffering effect was statistically significant but modest and weakened rather than eliminated the indirect association.

The study therefore supports a cautious interpretation of AI chatbot dependence as one statistical link within a broader pattern connecting social anxiety and resilience. Generative AI is theoretically distinctive because it combines instrumental utility with personalized quasi-social interaction, yet dependence on such systems should not be equated automatically with problematic use. Because reverse or reciprocal relationships remain plausible in cross-sectional data, future longitudinal and experimental research is needed to determine whether AI reliance is a precursor, consequence, correlate, or reciprocal component of psychological adaptation.

## Figures and Tables

**Table 1:** Demographic Characteristics of Respondents (N = 395).

Variable	Category	Frequency	Percentage (%)
Gender	Male	186	47.09
	Female	209	52.91
Age	18–25	49	12.41
	26–35	228	57.72
	36–45	91	23.04
	46 years or above	27	6.84
Education	High school or below	5	1.27
	College	29	7.34
	Bachelor’s degree	331	83.80
	Master’s degree	27	6.84
	Doctoral degree or above	3	0.76
Daily AI Chatbot Usage	Less than 30 min	71	17.97
	30 min–1 h	158	40.00
	1–2 h	120	30.38
	2–4 h	40	10.13
	More than 4 h	6	1.52
Geographic Region (IP-based)	Eastern China	223	56.46
	Central China	74	18.73
	Western China	73	18.48
	Northeastern China	23	5.82
	Overseas IP location	2	0.51

**Table 2:** Common Method Bias Assessment.

Test	Criterion	Result
Harman Single Factor	<50%	46.183%
Full Collinearity VIF	<3.3	1.698–2.650

**Table 3:** Reliability and Convergent Validity Result.

Variable	Items	Estimate	AVE	CR	α
SA	SA1	0.793	0.657	0.920	0.920
	SA2	0.849			
	SA3	0.796			
	SA4	0.794			
	SA5	0.809			
	SA6	0.822			
AICD	AICD1	0.756	0.545	0.905	0.905
	AICD2	0.810			
	AICD3	0.831			
	AICD4	0.702			
	AICD5	0.732			
	AICD6	0.649			
	AICD7	0.714			
	AICD8	0.694			
PSS	PSS1	0.681	0.567	0.940	0.939
	PSS2	0.768			
	PSS3	0.765			
	PSS4	0.643			
	PSS5	0.760			
	PSS6	0.767			
	PSS7	0.762			
	PSS8	0.746			
	PSS9	0.793			
	PSS10	0.755			
	PSS11	0.791			
	PSS12	0.789			
RES	RES1	0.827	0.664	0.952	0.952
	RES2	0.778			
	RES3	0.833			
	RES4	0.806			
	RES5	0.802			
	RES6	0.843			
	RES7	0.812			
	RES8	0.825			
	RES9	0.832			
	RES10	0.792			

**Table 4:** Item-Level Reliability Diagnostics.

Construct	Item	Corrected Item–Total r	α if Item Deleted
SA	SA1	0.752	0.908
SA	SA2	0.795	0.902
SA	SA3	0.761	0.906
SA	SA4	0.755	0.908
SA	SA5	0.784	0.903
SA	SA6	0.782	0.904
AICD	AICD1	0.679	0.894
AICD	AICD2	0.756	0.887
AICD	AICD3	0.779	0.885
AICD	AICD4	0.669	0.895
AICD	AICD5	0.699	0.892
AICD	AICD6	0.631	0.899
AICD	AICD7	0.693	0.893
AICD	AICD8	0.669	0.895
PSS	PSS1	0.659	0.936
PSS	PSS2	0.747	0.933
PSS	PSS3	0.744	0.933
PSS	PSS4	0.629	0.938
PSS	PSS5	0.738	0.934
PSS	PSS6	0.739	0.934
PSS	PSS7	0.728	0.934
PSS	PSS8	0.717	0.934
PSS	PSS9	0.768	0.932
PSS	PSS10	0.725	0.934
PSS	PSS11	0.765	0.933
PSS	PSS12	0.764	0.933
RES	RES1	0.800	0.946
RES	RES2	0.758	0.948
RES	RES3	0.808	0.946
RES	RES4	0.787	0.947
RES	RES5	0.780	0.947
RES	RES6	0.822	0.945
RES	RES7	0.790	0.947
RES	RES8	0.807	0.946
RES	RES9	0.810	0.946
RES	RES10	0.769	0.948

**Table 5:** Measurement Model Fit.

Fit Index	Reference Guideline	Result
χ^2^/*df*	<3.00	2.583
CFI	>0.90	0.914
TLI	>0.90	0.907
RMSEA	<0.08	0.063
SRMR	<0.08	0.047

**Table 6:** Fornell–Larcker Criterion.

Construct	SA	AICD	PSS	RES
SA	0.811			
AICD	0.691	0.738		
PSS	−0.623	−0.524	0.753	
RES	−0.767	−0.574	0.720	0.815

**Table 7:** HTMT Ratios.

Construct Pair	HTMT
SA—AICD	0.683
SA—PSS	0.622
SA—RES	0.767
AICD—PSS	0.521
AICD—RES	0.566
PSS—RES	0.721

**Table 8:** Means, Standard Deviations and Correlations.

Variable	Mean	*SD*	SA	AICD	PSS	RES
SA	3.165	1.390				
AICD	3.903	1.343	0.623 ***			
PSS	5.314	1.100	−0.578 ***	−0.479 ***		
RES	5.200	1.245	−0.718 ***	−0.525 ***	0.680 ***	

Note. *** *p* < 0.001.

**Table 9:** Regression-Based Path Estimates for H1–H3.

Path	*b*	*β*	*t*	*p*	LLCI	ULCI	*R* ^2^	*f* ^2^
SA → AICD	0.601	0.623	15.77	<0.001	0.5263	0.6762	0.388	0.633
SA → RES	−0.573	−0.640	−14.396	<0.001	−0.6509	−0.4945	0.526	0.529
AICD → RES	−0.117	−0.126	−2.843	0.005	−0.1981	−0.0361	0.526	0.021

**Table 10:** Indirect Association Analysis.

Association	Estimate	LLCI	ULCI
Total Association	−0.643	−0.718	−0.564
Direct Association	−0.573	−0.655	−0.489
Indirect Association	−0.070	−0.120	−0.022

**Table 11:** Hierarchical Regression Predicting AI Chatbot Dependence.

Step	Predictor	*b*	*SE*	*t*	*p*	*R* ^2^	Δ*R*^2^	Δ*F*
1	SA	0.601	0.038	15.770	<0.001	0.388		
2	SA	0.501	0.046	10.905	<0.001	0.409	0.021	14.089
	PSS	−0.218	0.058	−3.754	<0.001			
3	SA	0.489	0.046	10.598	<0.001	0.416	0.007	4.556
	PSS	−0.171	0.062	−2.759	0.006			
	SA × PSS	−0.066	0.031	−2.134	0.033			

Note. Continuous predictors were mean-centered before calculating the interaction term. ΔR^2^ and ΔF indicate changes relative to the immediately preceding step.

**Table 12:** Conditional Indirect Associations and Index of Moderated Mediation.

PSS Level	Estimate	LLCI	ULCI
Low	−0.066	−0.107	−0.022
Mean	−0.057	−0.094	−0.019
High	−0.049	−0.083	−0.015
Index of moderated mediation	0.008	0.001	0.016

## Data Availability

The data supporting the findings of this study are available from the corresponding author upon reasonable request. The data are not publicly available due to privacy considerations.

## Supplementary Materials

The following supporting information can be downloaded at: https://www.mdpi.com/article/10.3390/bs16091505/s1, Table S1. Demographic and Background Questions; Table S2. Measurement Items.

## Abbreviations

The following abbreviations are used in this manuscript:

SA	Social anxiety
AICD	AI chatbot dependence
PSS	Perceived social support
RES	Resilience
AICDS	AI chatbot dependence scale
AI	Artificial intelligence
SIAS-6	Six-Item Social Interaction Anxiety Scale
CD-RISC-10	10-Item Connor–Davidson Resilience Scale
