# Supplementary material for: AI Chatbot Dependence as an Indirect Link Between Social Anxiety and Psychological Resilience: The Moderating Role of Perceived Social Support
Source: Behav Sci (Basel). 2026 Aug 27;16(9):1505. doi: 10.3390/bs16091505 (PMC13603173; doi:10.3390/bs16091505)
Supplement: Supplementary file 1 [file behavsci-16-01505-s001.zip › behavsci-4521455-supplementary.pdf]

Table S1. Demographic and Background Questions.

Variable	Coding
Gender	1 = Male; 2 = Female
Age	1 = Under 18 (excluded from the analytic sample); 2 = 18–25 years old; 3 = 26–35 years old; 4 = 36–45 years old; 5 = 46 years or older
Educational level	1 = High school or below; 2 = Junior college; 3 = Bachelor's degree; 4 = Master's degree; 5 = Doctoral degree or above
Average daily AI chatbot usage	1 = Less than 30 minutes; 2 = 30 minutes–1 hour; 3 = 1–2 hours; 4 = 2–4 hours; 5 = More than 4 hours

Table S2. Measurement Items.

Construct	Code	Measurement Item	Source
Social Anxiety	SA1	I have difficulty making eye contact with others.	Peters et al. (2012)
	SA2	I find it difficult mixing comfortably with people.	
	SA3	I tense up if I meet an acquaintance in the street.	
	SA4	I am nervous mixing with people I do not know well.	
	SA5	I feel tense if I am alone with just one other person.	
	SA6	I have difficulty talking with other people.	
AI Chatbot Dependence	AICD1	If unable to use AI chatbots, I would feel anxious or uncomfortable.	Zhang et al. (2025)
	AICD2	I need to open AI chatbots before starting work or tasks.	
	AICD3	If unable to use AI chatbots, I would find it difficult to obtain the necessary information.	
	AICD4	Even when facing tasks or jobs that I could easily complete myself, I tend to seek assistance from AI chatbots.	
	AICD5	Compared with other people or things, I prefer to spend time on AI chatbots.	
	AICD6	Even if not actively using AI chatbots, I keep them logged in or running in the background.	
	AICD7	I am spending increasingly more time on AI chatbots.	
	AICD8	For me, life without AI chatbots would be inconvenient.	
Perceived Social Support	PSS1	My family really tries to help me.	Zimet et al. (1988)
	PSS2	I get the emotional help and support I need from my family.	
	PSS3	I can talk about my problems with my family.	
	PSS4	My family is willing to help me make decisions.	
	PSS5	My friends really try to help me.	
	PSS6	I can count on my friends when things go wrong.	
	PSS7	I have friends with whom I can share my joys and sorrows.	
	PSS8	I can talk about my problems with my friends.	
	PSS9	There is a special person who is around when I am in need.	
	PSS10	There is a special person with whom I can share my joys and sorrows.	
	PSS11	I have a special person who is a real source of comfort to me.	
	PSS12	There is a special person in my life who cares about my feelings.	

Construct	Code	Measurement Item	Source
Psychological Resilience	RES1	I am able to adapt when changes occur.	Campbell-Sills and Stein (2007)
	RES2	I can deal with whatever comes my way.	
	RES3	I try to see the humorous or positive side of things when facing problems.	
	RES4	Coping with stress can strengthen me.	
	RES5	I tend to bounce back after illness, injury, or other hardships.	
	RES6	I believe I can achieve my goals even when there are obstacles.	
	RES7	Under pressure, I stay focused and think clearly.	
	RES8	I am not easily discouraged by failure.	
	RES9	I think of myself as a strong person.	
	RES10	I can handle unpleasant or painful feelings.	
